# TRPV4—A Multifunctional Cellular Sensor Protein with Therapeutic Potential

**DOI:** 10.3390/s24216923

**Published:** 2024-10-29

**Authors:** Sanna Koskimäki, Sari Tojkander

**Affiliations:** Faculty of Medicine and Health Technology, Tampere University, 33520 Tampere, Finland; sari.tojkander@tuni.fi

**Keywords:** TRP channels, TRPV, TRPV4, sensors, mechanotransduction, therapeutic targeting

## Abstract

Transient receptor potential vanilloid (TRPV) channel proteins belong to the superfamily of TRP proteins that form cationic channels in the animal cell membranes. These proteins have various subtype-specific functions, serving, for example, as sensors for pain, pressure, pH, and mechanical extracellular stimuli. The sensing of extracellular cues by TRPV4 triggers Ca^2+^-influx through the channel, subsequently coordinating numerous intracellular signaling cascades in a spatio-temporal manner. As TRPV channels play such a wide role in various cellular and physiological functions, loss or impaired TRPV protein activity naturally contributes to many pathophysiological processes. This review concentrates on the known functions of TRPV4 sensor proteins and their potential as a therapeutic target.

## 1. TRPV4 Channel Proteins as Part of the TRP Channel Family

Transient receptor potential (TRP) channel proteins form a major group of calcium (Ca^2+^)-permeable channels, enabling rapid ion influx across the plasma membrane upon sensing diverse extracellular stimuli [1,2]. TRP channels can be activated, for instance by temperature changes, chemical compounds, oxidative stress, mechanical forces, or changes in the local physical environment of the cells. Consequently, they serve as universal sensors by detecting these various biophysical changes and subsequently regulating numerous physiological and pathological processes through downstream signaling pathways [3,4]. The TRP superfamily of proteins is composed of more than 30 different channel-forming proteins that are divided into seven subgroups: TRPV (vanilloid), TRPA (ankyrin), TRPC (canonical), TRPM (melastatin), TRPML (mucolipin), TRPN (NOMPC), and TRPP (polycystin) [5]. Each of these subgroup members has various, partially overlapping functions in distinct tissues.

The overall functions of the six TRPV (TRPV1–6) vanilloid-type proteins are highly diverse and subtype-specific. They can serve as sensors for different pain stimuli such as heat, pressure, and pH [6] but also contribute to the homeostasis of electrolytes, maintenance of barrier functions in multicellular sheets, as well as regulate the development of macrophages [6,7]. TRPV channels show species-dependent differences in their responses to various stimuli, and through their ability to respond to various extracellular cues, TRPV channels play a fundamental role in manifold physiological and pathophysiological processes, making them also promising targets for drug development [8].

Out of the TRPV family, TRPV4 is a polyselective cation channel that conducts monovalent as well as divalent cations, with a preference for Ca^2+^ [9,10]. The channel itself shows slight inward rectification at negative voltages and prominent outward rectification at highly positive voltages, while other TRPV channels exhibit different voltage-dependent activation properties. In comparison to other TRPV channels, TRPV4 differs in its activation stimuli and sensitivity to different types of mechanical activators and osmotic stress. It also displays different types of selectivity to ions, gating properties, and species-specific downstream responses [10,11].

TRPV4 is found widely in various human tissues but is predominantly expressed in the brain, kidney, bladder, skin, lung, heart, and gut (Table 1) [12]. While TRPV4 is mainly known for its functions at the cellular membrane, it is also expressed on the endoplasmic reticulum (ER) membrane of the cells, indicating its involvement in intracellular signaling pathways [13]. The ability of the channel to regulate calcium homeostasis and respond to different stimuli is crucial in maintaining various cellular functions and survival of the cells, as reviewed in the following chapters [12,14,15,16].

## 2. TRPV4 Functions in Epithelial Tissues

TRPV4-mediated signaling pathways regulate various physiological processes in the epithelium [38], from wound healing to epithelial–mesenchymal transition. The activation of the TRPV4 sensor has been quite extensively studied in respect of the epithelial permeability, consequently affecting the movement of ions and molecules across cell layers—an essential function for maintaining the ion homeostasis [39,40]. TRPV4-mediated signaling pathways can impact electrogenic ion flux and conductance rate f.i. through interactions with other signaling molecules and pathways, such as protein kinase C (PKC) and phosphoinositide 3-kinase (PI3K) [41]. Thus, TRPV4 is essential for the transepithelial ion flux, fluid secretion, and absorption in epithelial tissues [41]. The role of the TRPV4 sensor in epithelial tissues is introduced in more detail in the following chapters.

### 2.1. Skin Barrier and Wound Healing

TRPV4 has a crucial role in maintaining the integrity and permeability of the skin [42]. The activation of TRPV4 intricately modulates the barrier function of the skin, underscoring its significance in safeguarding the body against external damaging factors. Its activation serves as a gateway to tactile sensations, facilitating interactions with the environment and serving several key functions essential for skin homeostasis and sensory perception [36,37].

Activation of TRPV4 takes place in warm temperatures (27–35 °C) or in the presence of chemical agonists that initialize cell-cell junction development in the human skin keratinocytes [36,43]. In epidermal cell barriers, characterized by tight junctions, TRPV4 activation has been shown to upregulate occluding and claudin-4 proteins as well as protein kinase C (PKC), which are major factors contributing to intact tight junctions [44]. While it was initially identified as an osmolarity sensor, it is also involved in sensing osmotic changes in the skin microenvironment and responding to alterations in the osmotic pressure [11]. As demonstrated by fish models, osmotic stress is sensed through a TRPV4/Ca^2+^/transforming growth factor (TGF)-β–activated kinase 1 (TAK1)/nuclear factor kappa B (NF-κB) signaling pathway in the skin keratinocytes, which maintains osmotic homeostasis and mediates inflammatory responses [36,45]. TRPV4 also plays a role in cell differentiation and apoptosis, both essential for maintaining the skin barrier function [10,12,45].

Nociception (pain sensation) and itch perception in the skin are mediated by TRPV4 channels in keratinocytes and sensory neurons, with possible involvement of other cell lineages as well [36]. In the skin innervating sensory neurons, nociception is mediated by proteinase-receptor 2 (PAR-2) that can be activated by TRPV4 together with pro-inflammatory mediators such as prostaglandin E2, leading to heightened nociception of osmotic or mechanical stimuli [46]. Furthermore, TRPV4 expression in the fibroblasts and hair follicles is associated with collagen synthesis, indicating that TRPV4 is a possible regulator of skin repair and fibrotic processes in the aging and healing skin as well as in hair follicle function [36]. TRPV4 expression is elevated in the wounded area of the skin, further indicating its involvement in the wound healing process [47]. In mice studies using TRPV4 knock-outs, the lack of TRPV4 results in significantly suppressed re-epithelialization, formation of granulation in tissue, collagen deposition, and the number of α-SMA-positive myofibroblasts in skin wounds. In addition, the mRNA expression of collagen-production-related and myofibroblast-differentiation-related genes COL1A1 and ACTA2 is also decreased in the skin lesions of TRPV4 KO mice [48]. Additionally, in vitro studies using selective TRPV4 antagonists and agonists in cultures of keratinocytes and fibroblasts demonstrate TRPV4’s role in the regulation of cell migration and proliferation, further supporting its importance in wound healing [47,48].

Additionally, TRPV4 has been linked to various skin diseases, from atopic dermatitis to inflammatory conditions, highlighting its potential as a therapeutic target in such conditions [43,49]. In inflammatory conditions, TRPV4 activation can fuel cutaneous neurogenic and chronic inflammatory responses [37,50]. Together with receptors like TRPA1, TRPV4 contributes to the initiation and perpetuation of pro-inflammatory cascades when activated or sensitized [47]. For example, in a mouse model of keratinocytes with inhibited TRPV4, the mice were less sensitive to noxious stimuli and showed reduced inflammatory responses and secretion of inflammatory mediators, i.e., IL6 [51].

### 2.2. Other Epithelial Functions

In the mammary epithelium, TRPV4 acts as a mechanosensor and detects physical cues from the cell microenvironment, controlling ion influx in a spatio-temporal manner and converting biophysical signals from the exterior into biochemical signaling cascades within the cells [10,34]. As TRPV4 is especially prominently expressed in the stem/progenitor cell populations of the mammary epithelium, its functions in this epithelial cell type are likely associated with cell differentiation [10,34]. The expression of TRPV4 is also tension-dependent and impacts the differentiation process of the mammary stem/progenitor cells upon rigidity changes in the microenvironment [35]. Interestingly, TRPV4 itself has the ability to modulate properties of the biophysical environment by altering the expression of some extracellular matrix proteins [52]. Furthermore, depletion of TRPV4 impacts signaling pathways upstream of actomyosin assembly and contractility, therefore playing a role in cellular force production of the mammary epithelial cell layers and subsequently affecting their ability to maintain intact epithelial sheets and 3D morphology [10,34]. This suggests that TRPV4 acts as a critical player in the homeostasis of normal mammary epithelium by sensing the physical environment and guiding both differentiation of the mammary epithelial cell populations as well as the structural maintenance through cellular force production.

The activation of TRPV4 and hemichannels in the non-pigmented ciliary epithelium has significant implications for ocular health [27]. TRPV4 and hemichannels play a role in modulating aqueous humor secretion. Aqueous humor is essential for maintaining intraocular pressure and providing nutrients to the avascular tissues of the eye. Dysregulation of aqueous humor dynamics can lead to pathophysiological conditions such as glaucoma. [27] Additionally, TRPV4 activation in response to mechanical stimuli suggests a mechanosensitive function for these channels in non-pigmented ciliary epithelial (NPE) cells [28]. This mechanosensation ability may be crucial for the cells to respond to changes in intraocular pressure or other mechanical stresses within the eye. The cooperation between TRPV4 and hemichannels in response to swelling suggests a role in regulating cell volume, which is essential for maintaining cellular function and integrity, especially in a specialized tissue like the ciliary epithelium involved in fluid secretion [27]. The activation of TRPV4 and hemichannels leads to the release of ATP and possibly other signaling molecules. These molecules can act as autocrine or paracrine signals, influencing various cellular processes within the eye. [27] Additionally, TRPV4 channels have been linked to the maintenance of corneal homeostasis [4] and, in the retina, TRPV4 influences a variety of cell types, including ganglion cell soma-dendrite, microglia, and Müller cells, suggesting roles in common eye diseases like glaucoma, which is common in the age group of over 40 years old [53,54].

In the respiratory system, TRPV4 maintains osmotic pressure and homeostasis, regulating pulmonary artery relaxation, vasoconstriction, and the integrity of the alveolar epithelial barrier [32,33]. Other than its epithelial functions, TRPV4 also contributes to the functions in the osteoblasts and chondrocytes and participates in mechanotransduction within the skeletal system [19,31]. In the kidneys, TRPV4 contributes to the regulation of osmotic balance and water secretion, particularly in the distal convoluted tubule and regions where osmotic gradients develop [10]. In specific epithelial cells like those in the distal nephron of the kidney, TRPV4 channels are part of the process involved in potassium reabsorption. They respond to elevated flows and regulate intracellular calcium levels, contributing to overall renal function [14]. Finally, TRPV4 has been linked to the function of urothelial cells (UCs), layer, and regulation of urinary bladder voiding [17,18]. A study by Alpizar et al., 2020, showed that the activation of TRPV4 by gram-negative bacterial wall component lipopolysaccharides, LPS, in UCs, controls the pro-inflammatory responses and plays a role in the LPS-induced increase in voiding frequency. These discoveries provide evidence that the TRPV4 channel acts as a detector of bacterial LPS and facilitates rapid innate immune responses against gram-negative bacteria [17].

### 2.3. The Role of TRPV4 in Epithelial–Mesenchymal Transition

**Figure 1:**
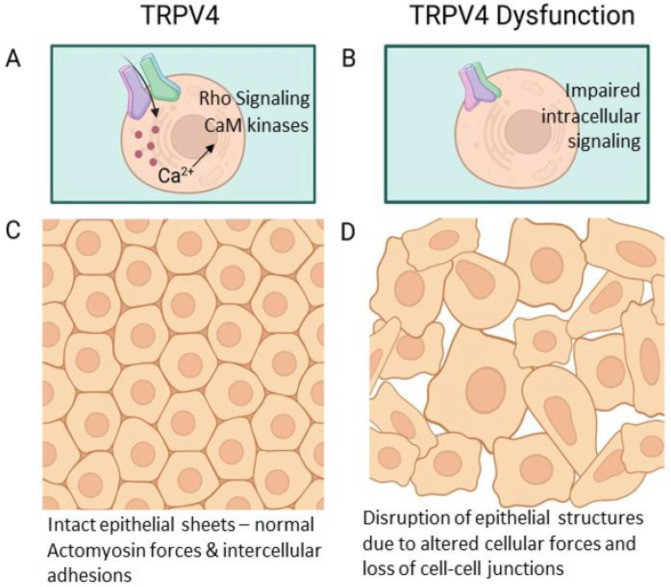
TRPV4 channel in the maintenance of epithelial integrity. (**A**) Spatially and temporally very strictly controlled ion influx through TRPV4 channel in response to several extracellular cues affects various cellular processes through its impact on intracellular cascades. One of these TRPV4-dependent functions is the maintenance of epithelial integrity through the control of actomyosin assembly. (**B**) TRPV4 dysfunction, namely over- or under-expression, can, therefore, have a major impact on the cellular functions within different tissue types through the disturbance in normal calcium influx. (**C**) In epithelial sheets, TRPV4 takes part in the control of intact epithelial junctions, maintaining epithelial integrity and permeability. This takes place at least through its impact on junctional proteins and actomyosin contractility, which is important for junctional force production. TRPV4 can regulate actomyosin contractility at least through the Ca^2+^/Calmodulin pathway and Rho kinases (**D**) Loss of TRPV4 from the epithelial cell populations subsequently leads to disrupted cellular features and impaired epithelial integrity, leading to mesenchymal cell types.

The epithelial–mesenchymal transition (EMT) is a process that is characterized by changes in the biochemical signaling cascades, leading to loss of epithelial cell polarity and cell-cell adhesions (see also Figure 1). This is associated with the transition into a mesenchymal cellular phenotype and the capacity to migrate and invade [55,56]. TRPV4-mediated calcium influx has been shown to be involved in the context of EMT [57,58,59], and hyperactivation of TRPV4 is associated with EMT in several cancer types, including breast, colorectal, and gastric cancers [60]. Furthermore, TRPV4-related calcium influx caused by hyperosmotic stress has been associated with the induction of EMT in tubular epithelial cell types [60]. Hyperosmotic stress can influence EMT induction through TRPV4-dependent signaling pathways upstream of the actin cytoskeleton [60].

In breast cancer cells, TRPV4 activation has significant implications on the EMT process through changes in the actin cytoskeleton and expression of EMT markers [34,35,61]. Interference of TRPV4 activation and levels leads to a more migratory phenotype, allowing cancer cells to acquire invasive and metastatic properties [34,35,61]. TRPV4 activation induces the expression of EMT markers such as vimentin, Snail, AXL, SERPINE1, and CD44 both in normal mammary epithelial and breast cancer cells [34,61]. These changes in gene expression and protein levels, associated with EMT, suggest that TRPV4 drives the transition of cancer cells toward a more aggressive phenotype and can even cause normal mammary epithelial cells to adopt a more migratory phenotype. Additionally, overexpression of TRPV4 has been shown to enhance the migration and invasion capabilities of colon cancer cells [58]. The role of TRPV4 in colon cancer development and progression takes place through the activation of the AKT signaling pathway and regulation of ZEB1 expression, consequently leading to EMT [58]. A similar phenomenon has been observed in hepatocellular carcinoma, where inhibition of TRPV4 has been shown to reduce the malignant progression of the carcinoma cells via the ERK signaling pathway [59].

The EMT process is also known to be influenced by the biophysical properties of the cell microenvironment and, interestingly, TRPV4 has been associated with the regulation of matrix stiffness and EMT through transforming growth factor β1 (TGFβ1) [57,58,62]. TRPV4 is involved in matrix stiffness-induced EMT and in the normal mouse primary epidermal keratinocytes (NMEKs). When NMEKs lacking TRPV4 are exposed to stiff matrices, they do not undergo EMT-like morphological changes and retain their epithelial morphology [62]. In contrast, wild-type NMEKs exhibit EMT-like changes when grown on stiff substrates, which can be further enhanced by the addition of TGFβ1 [62]. Additionally, TRPV4 antagonists inhibit wound closure and reduce the adhesive properties of cells grown on different stiffness ranges, indicating the critical regulatory role of TRPV4 in cell adhesion and migration on stiff matrices [63].

In summary, TRPV4 channels are versatile regulators of epithelial cell function, influencing ion transport, cell volume, sensory responses, and differentiation of the cells, thereby contributing to the physiological functions and disease progression. In cancers, TRPV4 acts as a critical regulator of both matrix stiffness and EMT, influencing cell migration capability and cancer progression. These functions highlight the diverse roles of the TRPV4 ion channel in various physiological systems and its potential as a therapeutic target for epithelial-related conditions, including various cancers.

## 3. Other TRPV4-Associated Functions in Various Tissue Types

### 3.1. Viral Entry

TRPV4 has been indicated to have a role in viral entry, partially through still unknown mechanisms. TRPV4 participates in the viral entry at least by recognizing some of the virus-produced proteins, subsequently triggering Ca^2+^-influx through the TRPV4 channel and activating specific intracellular pathways involved in the entry mechanisms of the virus [64,65,66]. Today, it is known that TRPV4 is involved in at least the Human papilloma, Ebola, Zika, and Herpes Simplex Virus infections. In the case of the Zika virus, activation of TRPV4 by viral proteins can lead to the nuclear translocation of DEAD-box RNA helicase DDX3X, which is involved in the nuclear export of viral RNAs and translation of viral proteins, including that of Zika virus (ZIKV) [64]. TRPV4 activation, followed by nuclear translocation of DDX3X, suggests the involvement of the Ca^2+^/CaM/CaMKII pathway [64]. On the other hand, in human vaginal epithelial cells, TRPV4 channel-mediated calcium influx facilitates viral entry during HSV (herpes simplex virus)-2 infection, and inhibition of the channel reduces the HSV infection and decreases inflammatory responses defined by expression of IL-6, tumor necrosis factor (TNF)-α, C-X-C motif chemokine ligand (CXCL)-9, and CXCL-10 [66]. In the infected cell membranes, TRPV4 channel protein directly interacts with HSV-2 glycoprotein D to enhance the viral infectivity. In line with the observations, inhibition of TRPV4 channel activity reduces inflammatory signals upon HSV-2 infection, indicating a strong association between TRPV4 and inflammatory responses in the epithelial cells [66]. Thus, inhibition of TRPV4 channels can have a dual impact, exhibiting both antiviral and anti-inflammatory properties through calcium signaling. In all, the above-mentioned findings suggest that TRPV4 channel activity may play a role in the early stages of viral entry and infection, and modulation of TRPV4 activity could also have therapeutic potential in viral diseases. Further research is, however, needed to fully understand the mechanisms by which TRPV4 contributes to viral entry to explore its potential as a target for antiviral therapies.

### 3.2. Programmed Cell Death

TRPV4 plays a significant role in programmed cell death through apoptosis, ferroptosis, necroptosis, pyroptosis, and autophagy [67,68,69]. Spatio-temporal influx of Ca^2+^ through TRPV4 channels can trigger different molecular events associated with cell death, such as endoplasmic reticulum (ER) stress, oxidative stress, and inflammation. This takes place through modulation of various apoptotic proteins, including CSP-3, CSP-9, Bax, and Bcl-2 [12]. Additionally, TRPV4 activation is linked to mitochondrial dysfunction, which can further contribute to apoptosis and neuronal cell death [70]. Additionally, in cardiomyocytes, TRPV4 has been linked to Ca^2+^-overload-linked hypoxic injury, while inhibition of TRPV4 has been shown to alleviate apoptosis by mitigating endoplasmic reticulum stress via PERK-eIF2α-ATF4-CHOP signaling pathway [71]. In general, inhibition of TRPV4 has been shown to decrease oxidative injury and cell death in other models as well, further highlighting the crucial role of TRPV4 in the regulation of programmed cell death processes in different cell types [70].

### 3.3. Regulation of Endothelial and Vascular Function

TRPV4 channels are found both in the endothelium and vascular smooth muscle cells, where they are influenced by different types of biophysical cues (e.g., shear stress, hydropic changes, heat) and chemical stimuli that regulate vascular tone [29]. In endothelial cells, activation of TRPV4 channels regulates calcium entry as a part of a so-called mechanosensitive membrane domain [72]. These mechanosensitive domains are characterized by the polarization of Caveolin-1-rich microdomains to the downstream end of the endothelial cells that are exposed to high laminar flow. While TRPV4 channels are ubiquitously expressed on the endothelial plasma membrane, they mediate only localized calcium entry, specifically at these microdomains where they physically interact with clustered Caveolin-1, leading to focal calcium bursts that activate endothelial nitric oxide synthase (eNOS) within these sites. TRPV4 participates in the endothelial function by regulating vascular tone and blood flow through vasodilator NO, produced by eNOS in response to various stimuli [30,31]. This is crucial for maintaining proper vascular tone and homeostasis [30,31]. Spatially restricted activation of eNOS in the domains is essential for the anti-inflammatory phenotype observed in arterial endothelial cells that meet high laminar flow [32]. Furthermore, the mechanosensitive nature of these domains is highlighted by the fact that calcium oscillations are sustained only in the presence of laminar shear stress, further suggesting that this signaling activity is dependent on mechanical forces [73]. It has also been shown that Vascular smooth muscle cell (VSMC) proliferation and migration is decreased in the presence of NO, and therefore, TRPV4-mediated NO production may prevent excessive VSMC proliferation that can be associated with vascular remodeling and –diseases [31].

Interestingly, Angiotensin II (Ang II) treatment significantly reduces TRPV4 protein expression in endothelial cells [74]. Ang II takes part in the regulation of blood pressure by inducing smooth muscle cell contraction and vascular remodeling through binding to G protein-coupled receptor (GPCR) AT1R, subsequently activating G_q_/PLC/IP_3_ mediated Ca^2+^ influx [75,76]. Ang II inhibits TRPV4-mediated Ca^2+^ influx in human endothelial cells without altering TRPV4 mRNA levels, suggesting that Ang II may directly interfere with the activity of TRPV4, eventually leading to reduced calcium influx even in the presence of a proper activator [74]. Interestingly, Ang II has been shown to crosstalk with TRPV4 via the angiotensin 1 receptor (AT1R) and β-arrestin signaling pathway in epithelial and immortalized cell types [77]. This crosstalk may also involve downstream signaling cascades that ultimately result in the downregulation of TRPV4 channels in endothelial cells, suggesting at least two distinct pathways for TRPV4 interference [74,77]. Ang II-mediated downregulation of TRPV4 channels can also affect TRPV4-induced eNOS phosphorylation and NO production in endothelial cells [74]. This disruption in the TRPV4/eNOS/NO signaling axis can contribute to endothelial dysfunction and vascular remodeling [74].

TRPV4 channels are also found in the endothelial and epithelial cells of the lungs, where they regulate endothelial permeability and integrity of the lung tissue [45,78]. However, in the context of lung injury, TRPV4 channels have been implicated in the induction of endothelial permeability, leading to the disruption of the alveolar barrier function and the accumulation of protein-rich fluid in the alveolar space [32]. The disruption can result in life-threatening conditions such as pulmonary edema, acute lung injury (ALI), and acute respiratory distress syndrome (ARDS). Additionally, TRPV4 channels have been associated with ventilator-induced lung injury and lung ischemia/reperfusion injury through pulmonary endothelial barrier disruption [79]. Other than the alveolar barrier function, activation of TRPV4 channels in various cell types within the lungs, including alveolar epithelial and endothelial cells, as well as neutrophils, has been linked to increased reactive oxygen species (ROS) production [32]. In summary, TRPV4 channels contribute to lung injury by promoting endothelial permeability, barrier disruption, edema formation, and inflammatory responses, which can lead to severe lung conditions.

Furthermore, TRPV4 functions as a microenvironment sensor that can affect the formation of edema through its interplay with aquaporin-4 (AQP4) [80]. The study by Nishinaka et al. shows that TRPV4 expression in the retinas of the eye in RVO mice (Retinal Vein Occlusion mouse model) is especially prominent in the ganglion cell layer (GCL) [81]. This increased expression of TRPV4 is associated with the aggravation of ischemic conditions and the development of retinal edema in the RVO model. Furthermore, TRPV4 is closely associated with increased vascular permeability in retinal blood vessels, leading to increased retinal edema and ischemia [81]. In all, the findings suggest that TRPV4 channels are involved in the regulation of vascular permeability in the retina as well and that targeting TRPV4 with antagonists could provide a new therapeutic approach for treating retinal vascular diseases through inhibition of inflammatory factors and aquaporin-4 (AQP4).

### 3.4. Nervous System and TRPV4 Sensor Protein

Expression of TRPV4 in sensory afferent neurons has been linked to inflammatory and neuropathic pain [20]. In line with that, TRPV4 antagonists and knockdown of TRPV4 levels reduce nociception in chemotherapy-induced peripheral neuropathy (CIPN) [21,22]. Further on, enhanced expression of TRPV4 receptors has been observed in cancer-induced bone pain (CIBP), perineural, and orofacial cancer models, indicating the involvement of TRPV4 in the sensation of cancer pain [82]. A key mechanism suggested in CIPN depends on the altered expression and functional disruption of TRP channels, including the TRPV4 sensor. Antineoplastic drugs (i.e., paclitaxel, thalidomide) can intensify the function of TRPV4, leading to hypotonic-induced nociception [21,22,83]. In addition to CIPN, various neuropathic pain models underscore TRPV4’s multifaceted involvement [82].

In addition, elevated TRPV4 expression induces endoplasmic reticulum (ER) stress and neuroinflammation [84]. More specifically, activation of TRPV4 downregulates sarco/endoplasmic reticulum Ca^2+^-ATPase 2 (SERCA2) expression and upregulates ER stress-associated proteins like GRP78, GRP94, CHOP, and procaspase-12, supporting their expression within the inflammatory milieu [85]. Additionally, TRPV4 is implicated in the initiation of Ca^2+^-induced Ca^2+^ release (CICR) via the ryanodine receptor and inositol (1,4,5)-trisphosphate receptor, further intensifying ER stress. Knockdown of TRPV4 has been demonstrated to alleviate ER stress by reducing CHOP expression and procaspase-12 activation, suggesting its potential as a therapeutic target for neuroinflammatory conditions, including Parkinson’s disease [85].

Moreover, the effects of TRPV4 hyperactivation extend to dysfunction of the blood-brain barrier (BBB) [23,24]. TRPV4 activation can drive the endothelium to a pro-inflammatory state, leading to increased expression of cell adhesion molecules and pro-inflammatory cytokines that maintain local endothelial inflammation [23,25]. TRPV4 upregulation in inflamed endothelial cells, therefore, accelerates BBB dysfunction, leading to increased permeability and loss of junctional integrity, favoring the migration of immune cells into the central nervous system [25]. Furthermore, TRPV4 mediates the expression of E-selectin in the extravasating immune cells across the BBB [25]. In line with this, inhibition of TRPV4 reduces E-selectin expression and leads to decreased migration of T cells across the inflamed brain endothelial cells. In addition, TRPV4 activity is involved in calcium-driven cytoskeletal remodeling that is essential for leukocyte transmigration [86]. The inhibition of TRPV4 reduces T cell migration across the BBB, suggesting its direct role in the T cell migration process. Lastly, TRPV4 activation stimulates the NFκB pathway and promotes the transition of endothelial cells into a pro-inflammatory phenotype, which contributes to BBB dysfunction and neuroinflammation that is typical for multiple sclerosis (MS) [25].

Interestingly, while elevated TRPV4 expression contributes to BBB dysfunction, mutant TRPV4 expression in endothelial cells causes focal breakdown of the blood-spinal cord barrier (BSCB), leading to disruptions in BSCB integrity [26]. Mutant TRPV4 channels exhibit a gain of function in their activity in neural vascular endothelial cells (NVECs), resulting in a rapid decline in barrier integrity after the activation of TRPV4 channels. The focal disruptions in BSCB integrity in symptomatic TRPV4 mutant mice are associated with motor behavioral impairments, regional motor neuron loss, and early lethality [26]. TRPV4 is also involved in the development of diabetic retinopathy due to its role in the regulation of membrane potential and responses to osmotic pressure in the retinal neurons [87]. The activation of TRPV4 under high intraocular pressure can induce Müller cell gliosis and release of tumor necrosis factor-α (TNF-α). TRPV4 knock-out or inhibition shows promise in preventing diabetic retinopathy complications, again showing the potential of TRPV4 targeting in therapeutic approaches [88].

### 3.5. TRPV4 and Immune System

TRPV4 has additionally been found to play a role in the immune system. It is generally involved in modulating the activity of the key immune cells, such as neutrophils, T cells, dendritic cells, and macrophages, which are all essential for combating infections and maintaining the homeostasis of different tissues [89,90,91]. Recent investigations have shown that TRPV4 plays a pivotal role in the mechanosensation of many immune cells, and through this activity, it could actually regulate the innate host defense responses, depending on the physical features of the microenvironment [92]. Mechanosensing through TRPV4 has been associated, for instance, with the adhesion, migration, and extravasation of the immune cells. TRPV4 can also stimulate in these cells many intracellular signaling pathways, such as MAPK (p38, JNK), YAP/TAZ, EDN1, NF-kB, and HIF-1α, to modulate gene expression for driving cytokine production, cellular survival, and proliferation [93]. Additionally, it has a direct impact on RhoGTPases, subsequently impacting cell adhesion and migration through integrins and actomyosin contractility. The role of TRPV4 in regulating cell maturation and migration has been studied, for instance, in dendritic cells where TRPV4 is essential for the immune response of this cell type [94]. In addition, in macrophages, TRPV4 activation is known to influence cell polarization and cytokine production [93]. TRPV4 activation in macrophages inhibits NF-κB signaling, leading to the suppression of IL-1β production that is involved in the development of inflammatory diseases such as atopic dermatitis [93]. TRPV4 also inhibits the differentiation of human primary monocytes into specific macrophages, suggesting that it has the potential to act as an anti-inflammatory target in this immune cell type. Furthermore, TRPV4 has been associated with the activation and migration of T cells, suggesting that it also regulates adaptive immune responses [95]. Inhibition of TRPV4 was shown to modulate the activation of T cells and their production of effector cytokines by suppressing the release of tumor necrosis factor, interleukin-2, and interferon-γ. In all, TRPV4 seems to have an important modulatory role both in the innate and adaptive immune systems. Further research is, however, needed to fully understand the mechanisms by which it influences various immune cell functions. Understanding these mechanisms in detail could lead to the development of novel therapeutic approaches for various immune-related disorders.

## 4. Modulators of TRPV4

As discussed in the above sections, TRPV4 has various physiological functions, and its impaired activity can have severe consequences. Several modulators of TRPV4 activity exist already, and will be discussed in the following chapters.

### 4.1. Activators

Lysophospholipids, more specifically lysophosphatidic acid (LPA), have been identified as an endogenous activator of TRPV4 channels [96]. The activation of TRPV4 by LPA occurs in a dose-dependent manner and involves specific residues in the N- and C-termini of the ion channel. LPA can induce larger single-channel currents through TRPV4, leading to increased channel conductance and cell excitability [97]. Other agonists for TRPV4 include 4α-phorbol 12,13-didecanoate (4αPDD) and GSK1016790A [98]. In addition, Ouabain is a well-known cardiac glycoside that is known for its role in inhibiting Na^+^/K^+^-ATPase and possessing the ability to modulate TRPV4 channels [14,99]. Upon binding to Na^+^/K^+^-ATPase, Ouabain activates several signaling pathways, including RAS/RAF/MEK/ERK and PI3K/Akt/mTOR. These pathways are crucial for both the short-term modulation of TRPV4 activity and the long-term promotion of new channel synthesis [16].

### 4.2. Inhibitors

TRPV4 antagonists are small molecules that inhibit the activity of TRPV4 channels. Largely, these antagonists bind to the voltage-sensing-like domain of TRPV4, stabilizing the channel in a closed state to block its activity [16]. Antagonists for the TRPV4 ion channel are being explored for their therapeutic potential in various conditions.

Non-selective TRPV4 inhibitors have been discovered already several years ago, and these include gadolinium and ruthenium red, which also have multiple effects beyond TRPV4 inhibition. Later, more selective TRPV4 antagonists have been developed, including compounds like RN-1734, HC-067047, and GSK2193874, which specifically target TRPV4 without affecting other channels [100,101]. Some of these have also shown efficacy in preclinical studies for conditions like pulmonary edema induced by heart failure [98].

Regarding vascular permeability and inflammation, RQ-00317310 is a novel TRPV4 antagonist that shows promising results in retinal edema and ischemia in RVO mice [74]. This compound was found to decrease the upregulation of TNF-α and the downregulation of AQP4 in the retina, leading to reduced inflammatory responses and maintenance of barrier integrity. Other TRPV4 antagonists, HC-067047, GSK2193874, and GSK2798745, have also demonstrated positive effects in improving retinal edema and ischemia in RVO mice, being able to inhibit the VEGF-induced vascular hyperpermeability [74]. Of these, GSK2798745 is a potent selective inhibitor of the TRPV4 channel that prevents channel activation and reduces pulmonary edema in heart failure patients through modulation of calcium influx [102,103,104]. GSK2798745 has advanced to Phase I clinical trials (NCT02119260), indicating progress toward potential clinical use in humans [98].

Furthermore, capsaicin affects TRPV4 channels by blocking Ca^2+^ entry through the channels [99]. Capsaicin is known to inhibit intestinal Cl^−^ secretion via blocking epithelial TRPV4 channels. This inhibition occurs via the suppression of TRPV4 channels by phosphatidylinositol 4,5-bisphosphate (PIP2), which can be relieved after the hydrolysis of PIP2 by phospholipase C (PLC) and phospholipase A2 (PLA2) during cholinergic signaling. Additionally, capsaicin dose-dependently inhibits Ca^2+^ entry via TRPV4 channels in intestinal epithelial cells. These findings suggest that capsaicin can modulate the activity of TRPV4 channels, potentially contributing to its effects on intestinal ion transport and colitis. [99]. In addition to the above, TRPV4 antagonist is the 2′,4′-dimethyl-[4,5′-bithiazol]−2-yl amino derivative (Antagonist 2, A2), developed for pain treatment [16].

The finding and development of several TRPV4 activators and inhibitors has greatly advanced our understanding of the functions of TRPV4 channel proteins but has also opened avenues for potential therapeutic applications in various conditions where TRPV4 channels play a pivotal role. Future research should aim in elucidating the precise molecular mechanisms behind these compounds, possible interactions with other channel proteins, and the potential for therapeutic use.

## 5. Cooperation of TRPV4 and Piezo1 Channels

Piezo1 is a piezo-type mechanosensitive ion channel protein that plays a role in epithelial integrity and has a significant influence on the activity of TRPV4 protein in various cell types, including trabecular meshwork cells, vascular endothelial cells, and osteoblasts [105,106,107]. The cooperation of Piezo1 and TRPV4 has been studied, for example, in the pancreatic stellate cells, PSCs, that play a key role in creating the rigid tumor tissue found in the pancreatic ductal adenocarcinoma (PDAC) [106]. PSCs establish a stiffness gradient between the healthy pancreas and the tumor, which triggers durotaxis, a type of cell movement that is influenced by varying stiffness levels. TRPV4 and Piezo1 seem to act as sensors, cooperating during the durotaxis. The cooperation of these channels has also been investigated in the trabecular meshwork cells (TMCs) that possess both endothelial and myofibroblast characteristics and play a crucial role in renewing the extracellular matrix (ECM) [108]. Primary TMCs express high levels of both Piezo1 and TRPV4, which are responsible for initiating remodeling of the ECM and cytoskeleton in a mechanosensitive manner. Based on this study, performed with the help of different modulators of TRPV4 and Piezo1, Piezo1 acts upstream of TRPV4 and can induce controlled opening of the TRPV4 channels, eventually resulting in elevated intracellular calcium levels [108]. In endothelial cells, Piezo1 regulates the activation of TRPV4 channels in response to fluid shear stress [108,109]. More specifically, Piezo1 can stimulate the opening of the TRPV4 channels through the activation of phospholipase A2, leading to sustained elevation of intracellular calcium levels that are responsible for fluid shear stress-mediated disruption of the adherent junctions and subsequent cytoskeletal remodeling [109]. Piezo1 and TRPV4 channels can, therefore, be functionally coupled in different cell types. On the other hand, a study performed with a hepatic portal vein that transports blood from the intestines, gallbladder, pancreas, and spleen to the liver did not show significant cooperation of Piezo1 and TRPV4 under mechanical and osmotic variations [105].

Other than the exposure of endothelial cells to shear stress, Piezo1 and TRPV4 are also known to cooperate in mechanotransduction in other situations, where Piezo1 mediates rapid Ca^2+^ currents in response to mechanical signals, while TRPV4 channels respond to stretch-induced activation of Piezo1 and regulate cellular functions further. One example of the functional coupling of TRPV4 and Piezo1 is in endothelial-dependent hyperpolarization (EDH)-mediated vasorelaxation [110]. In vascular endothelial cells, the activation of the Piezo1 channel initiates a calcium signaling that leads to the opening of TRPV4 channels. This, in turn, induces a further calcium entry that activates IKCa and SKCa channels in vascular endothelial cells (VEC), resulting in hyperpolarization and attenuation of calcium entry via VGCC in vascular smooth muscle cells (VSMC). Additionally, the increase in extracellular potassium between VEC and VSMC activates a coupling of NKA and NCX to further decrease cytoplasmic calcium concentration in VSMC. This functional coupling of Piezo1 and TRPV4 channels thus sustains endothelial calcium elevation and vasorelaxation of resistance vessels via the calcium/EDH pathway [110].

In summary, Piezo1 plays a key role in regulating the activation and function of TRPV4 channels in response to mechanical stimuli in certain cell types, highlighting the close interplay between these two mechanosensitive channels in cellular signaling pathways [108,109].

## 6. Discussion and Conclusions

**Figure 2:**
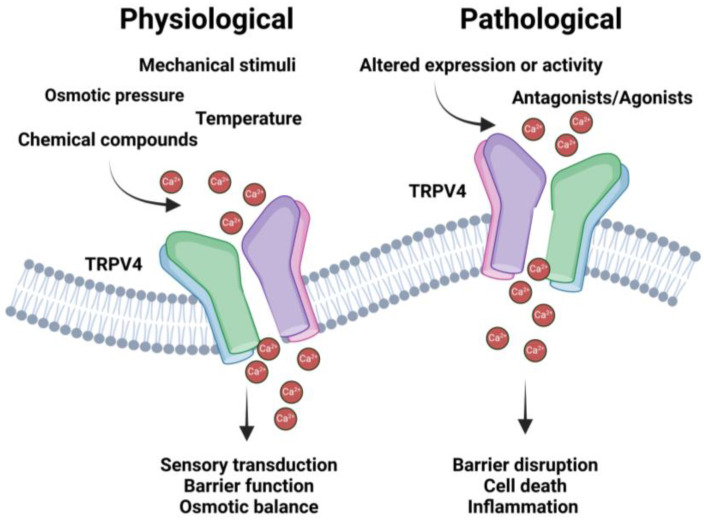
TRPV4 acts as an essential sensor of extracellular cues, such as mechanical stimuli, osmotic pressure, and temperature. Spatio-temporal activation of TRPV4 by these various cues leads to activation of intracellular signaling cascades through controlled calcium influx, therefore impacting several cellular events and homeostasis of distinct tissues. Altered TRPV4 activity is consequently associated with a number of pathophysiological conditions through deregulation of cellular functions.

TRPV4 has been widely studied in the maintenance of epithelial integrity and wound healing, as well as endothelial and vascular function. In addition, several studies have shown the multifaceted functions of this channel protein in sensory perception, immune cell function, virus entry, cell differentiation, and survival Figure 2.

Moreover, TRPV4 has been linked to various pathological processes, such as pain, inflammation, cancer, and cardiovascular conditions, and there is wide interest in investigating it for drug development [111]. Targeting TRPV4 in several pathological conditions may, therefore, have clinical relevance. Further studies are, however, needed to reveal the exact molecular mechanisms and cooperative pathways that are involved in the modulation of TRPV4 activity in different tissues. As discussed above, recent research has already shown some promising progress in utilizing TRPV4 as a therapeutic target, and scientists have identified many potential small molecule compounds that can be used for modulating TRPV4 activity [16]. These advances in the field can support research related to the therapeutical targeting of TRPV4 in conditions like osteoarthritis, pulmonary edema, neurogenic inflammation, or any other TRPV4-linked diseases. There have been great advances in targeting TRPV4 channels in challenging, chronic pain conditions, including cancer-associated pain [78]. In addition, TRPV4 antagonists have been shown to prevent pulmonary edema associated with heart failure in animal experiments, suggesting that there could be potential for clinical trials. For that, it has also been proposed that TRPV4 inhibitors could have relevance in treating COVID-19 patients who are at a high risk of developing lung edema [112]. In general, TRPV4 targeting has great potential in many chronic respiratory conditions, including asthma, chronic obstructive pulmonary disease (COPD), and chronic cough [113,114]. Research has shown that TRPV4 activity is elevated in lung fibroblasts that have been isolated from individuals with Idiopathic Pulmonary Fibrosis. In line with this observation, inhibition of TRPV4 activity also shows promise as a potential therapeutic strategy to treat this condition [115]. Furthermore, it will be interesting to follow how the TRPV4 targeting in immunological conditions and cancer progression will advance in the future. Understanding TRPV4’s function and the development of TRPV4-targeted compounds has definitely opened up new possibilities for therapeutic interventions in various diseases. As the regulation of this protein is so complex, further research, together with clinical trials, is clearly crucial for advancing the potential use of TRPV4 as a therapeutic target in various contexts and tissue types.

In conclusion, this review underscores the pivotal role of the TRPV4 ion channel as a cellular sensor in various physiological processes and in a wide range of tissues. Moreover, targeting TRPV4 in several pathological conditions may have clinical relevance and further studies will be needed for revealing the exact molecular mechanisms and cooperative pathways that are involved in modulating TRPV4 in several diseases.

## Figures and Tables

**Table 1:** TRPV4 expression and functions in different tissues.

Tissue Type	Function(s)
Bladder	Regulation of bladder voiding [17,18]
Bone	Mechanotransduction [19]
Brain	Pain perception, maintenance of blood-brain barrier [20,21,22,23,24,25,26]
Eye	Regulation of aqueous humor secretion and osmotic pressure [27,28]
Gut	Mechanosensation [12]
Heart and vascular system	Regulation of vascular tone and permeability and blood flow [29,30,31]
Kidney	Regulation of osmotic balance and water secretion [10,14]
Lung	Regulation of pulmonary artery relaxation, vasoconstriction, and the integrity of the alveolar epithelial barrier [32,33]
Mammary epithelium	Mechanosensation [34,35]
Skin	Maintenance of integrity and permeability of the skin, sensory perception [36,37]
